# Shorter versus longer-duration antibiotic treatments for immunocompetent patients with bloodstream infections: a Bayesian perspective

**DOI:** 10.1016/j.eclinm.2025.103673

**Published:** 2025-11-24

**Authors:** Ruslan Akhmedullin, Abduzhappar Gaipov

**Affiliations:** Department of Medicine, Nazarbayev University School of Medicine, Astana, Kazakhstan

We read with great interest the meta-analysis published by Liu et al.,^1^ which presented comparative findings on the efficacy and safety of shorter versus longer duration of antibiotic treatment for immunocompetent patients with bloodstream infections. We suggest that the interpretation of the findings be improved to strengthen their implications.

The authors claimed that “There are probably little or no differences” in mortality (RR 0.91, 95% CI 0.79–1.05) and relapses (RR 1.14, 95% CI 0.80–1.62) when comparing the treatment regimens. Any scientific statement should include a probability that expresses confidence in the effect.^2^ Hence, we suggest looking at the estimand from a Bayesian perspective.

We conducted Bayesian meta-analyses using weakly informative priors with a location of 0 and a scale of 1 in R-software. Such a prior distribution enables a stable estimation, allowing the data to dominate without being overly restrictive. The meta-analysis of mortality revealed similar estimates (RR 0.91, 95% CrI 0.79–1.06). Furthermore, we found 87.5% probability of RR being less than the null value (i.e., RR < 1.0). Alternatively, for relapses, we also observed an effect size similar to the (original) frequentist estimates (RR 1.14, 95% CrI 0.79–1.64). Admittedly, the probability of the posterior distribution being less than 1.0 was lower–22.7%. Hence, for mortality, the results favored the short regimen. For relapses, however, it was associated with increased risk. Additionally, we assessed the level of superiority by setting probability values across several RR thresholds. We found 41.5% probability of ≥10% mortality reduction and a 54.6% probability of ≥10% increase in relapses with the short regimen.

We believe that the Bayesian approach improves communication and offers more clinically relevant information when interpreting the findings.

## Contributors

Abduzhappar Gaipov: conceptualized, reviewed and edited the original draft. Ruslan Akhmedullin: Writing original draft. All authors read and approved the final version of the manuscript.

## Declaration of interests

We declare no competing interests.
